# Identification of Novel FNIN2 and FNIN3 Fibronectin-Derived Peptides That Promote Cell Adhesion, Proliferation and Differentiation in Primary Cells and Stem Cells

**DOI:** 10.3390/ijms22063042

**Published:** 2021-03-16

**Authors:** Eun-Ju Lee, Khurshid Ahmad, Shiva Pathak, SunJu Lee, Mohammad Hassan Baig, Jee-Heon Jeong, Kyung-Oh Doh, Dong-Mok Lee, Inho Choi

**Affiliations:** 1 Department of Medical Biotechnology, Yeungnam University, Gyeongsan 38541, Korea; gorapadoc0315@hanmail.net (E.-J.L.); ahmadkhursheed2008@gmail.com (K.A.); Sunju.lee@connext.co.kr (S.L.); mohdhassanbaig@gmail.com (M.H.B.); 2 Research Institute of Cell Culture, Yeungnam University, Gyeongsan 38541, Korea; 3 College of Pharmacy, Yeungnam University, Gyeongsan, Gyeongbuk 38541, Korea; shivapat@stanford.edu (S.P.); jeeheon@yu.ac.kr (J.-H.J.); 4 Department of Physiology, College of Medicine, Yeungnam University, Daegu 42415, Korea; kodoh@ynu.ac.kr; 5 Technology Convergence R&D Group, Korea Institute of Industrial Technology, Yeongcheon 770200, Korea; cowboyle@kitech.re.kr

**Keywords:** extracellular matrix, fibronectin, FNIN, mesenchymal stem cells, cell adhesion, proliferation, differentiation

## Abstract

In recent years, a major rise in the demand for biotherapeutic drugs has centered on enhancing the quality and efficacy of cell culture and developing new cell culture techniques. Here, we report fibronectin (FN) derived, novel peptides fibronectin-based intergrin binding peptide (FNIN)2 (18-mer) and FNIN3 (20-mer) which promote cell adhesion proliferation, and the differentiation of primary cells and stem cells. FNIN2 and 3 were designed based on the in silico interaction studies between FN and its receptors (integrin α5β1, αvβ3, and αIIbβ3). Analysis of the proliferation of seventeen-cell types showed that the effects of FNINs depend on their concentration and the existence of expressed integrins. Significant rhodamine-labeled FNIN2 fluorescence on the membranes of HeLa, HepG2, A498, and Du145 cells confirmed physical binding. Double coating with FNIN2 or 3 after polymerized dopamine (pDa) or polymerized tannic acid (pTA) precoating increased HBEpIC cell proliferation by 30–40 percent, suggesting FNINs potently affect primary cells. Furthermore, the proliferation of C2C12 myoblasts and human mesenchymal stem cells (MSCs) treated with FNINs was significantly increased in 2D/3D culture. FNINs also promoted MSC differentiation into osteoblasts. The results of this study offer a new approach to the production of core materials (e.g., cell culture medium components, scaffolds) for cell culture.

## 1. Introduction

Cell culture technology is a widely used research tool in basic biological science and biomedical research fields. In concert with the rapid development of methods of producing biopharmaceuticals (e.g., antibodies, proteins, vaccines, and cell therapeutics) by cell culture, tremendous efforts are being made to improve the efficiency of cell cultures and to develop new cell culture techniques. Cells exhibit many robust behaviors, such as matrix binding, migration, proliferation, and differentiation by constantly communicating with the external environment. In this context, the extracellular matrix (ECM) is known to control cell behavior through direct or indirect cell interactions [1,2].

In suspension cell cultures, which are frequently used for biopharmaceutical production, it is important to increase cost-effectiveness by reducing cell culture material requirements. In the cell therapy field, scientists are still confronted by problems, such as the tardy growth of cells isolated from a patient or alterations in the original characteristics of cells. For example, in the embryo stem cell field, it is difficult to maintain cell characteristics unless human embryonic stem cells are grown on top of feeder cells derived from either mouse or man [3,4]. Three-dimensional (3D) cell culture, which has become popular due to its similarity to in vivo systems, is difficult to achieve without a supply of ECM materials [5,6], but the complex structures of ECM proteins caused by post-translational modifications make the production of ECM proteins with consistent biological activity extremely challenging [7,8].

Peptides are “small molecules” composed of at least two amino acids (AAs) and have a broad variety of biological activities. Hypothetically, peptide therapy could provide a wealth of small biotherapeutics as the architectures of peptides are highly consistent, which makes them suitable for targeting proteins [9]. Peptides have major benefits over small molecules, such as high specificities, biological activities, and membrane permeation efficiencies, and low cost [10,11], and thus, the design of peptides that mimic specific binding protein sites has great therapeutic potential. Mimetics are typically built based on the 3D structures of protein complexes [12,13], which are the primary source of active peptides, as peptide fragments originating from protein–protein interactions are the key sources of rational drug design [14,15].

**Figure 1:**
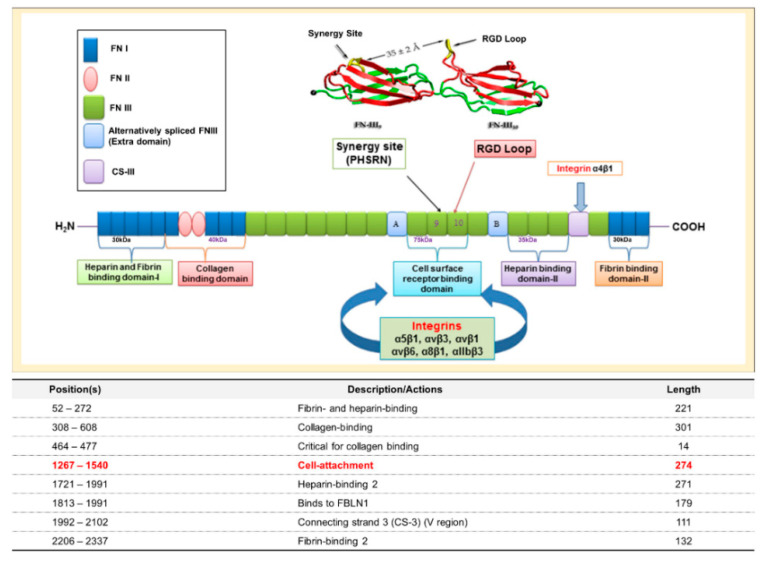
Structure and binding domains of fibronectin (FN). FN consists of three types of repeats FNI, FNII, and FNIII. These sets of repeats possess several binding domains viz. heparin, fibrin, collagen, and cell surface receptor-binding domains. Extra domains A and B are formed in alternatively spliced FNIII. CS-III is another alternatively spliced section and a binding site for integrin α4β1. The cell-attachment domain (from position 1267–1540) is the binding site for several integrins (e.g., α5β1, αvβ3, and αIIbβ3). The RGD loop and synergy site (PHSRN) is a key binding site for several integrins.

Fibronectin (FN) is a multifunctional glycoprotein that is widely distributed in many tissues. FN is a key component of the ECM and a potential ligand of the surface receptors of most cell types. Integrins are the foremost cell surface receptors and many (viz. α2β1, α3β1, α4β1, α4β7, α5β1, α8β1, αvβ1, αvβ3, αvβ5, αvβ6, αvβ8, and αIIbβ3) are known to bind FN [16,17,18,19] (Figure 1). These FN-integrin interactions result in many biological processes including cell adhesion, growth, migration, and differentiation. In particular, integrin α5β1 is a classical FN receptor and their interaction has been reported to initiate bidirectional (inside–out and outside–in) signaling pathways essential for cell differentiation, proliferation, and migration [20,21,22].

Several studies have shown that the FN cell-binding region (1267–1540), which contains the Arg-Gly-Asp (RGD) sequence, the most common peptide motif that binds with multiple integrin receptors [19,23,24], is crucial for the integrin binding that promotes cell adhesion, proliferation, and signaling pathways [25,26]. The present study was conducted with the aim of developing novel short peptides (FN derived) that are stable in cell culture and have the the potential to improve cell culture media quality and are easy to handle due to their small size and can therefore be used as ECM mimetic substances. We selected the FN cell-binding region to design short mimetic peptides based on integrin and FN protein tertiary structures known to be involved in ECM/cell communication in almost all animal cells.

## 2. Results

### 2.1. Interaction of Fibronectin with Integrin Receptors

The binding efficacy of FN–integrin was assessed using global energies predicted by FireDock. The global energies of interactions between integrins (α5β1, IIbβ3, and αvβ3) and FN were found to be −58.51 and −40.19, and −25.27 respectively (Table 1a,b).

### 2.2. Interactions between FNIN2 and FNIN3 and Integrin Receptors

The global energies of fibronectin-based intergrin binding peptide (FNIN)2 or 3 bindings with integrin receptors are listed in Table 1b. The binding efficacy is considered best for lower global energies (binding energies), i.e., larger negative values. The global energies for FNIN2 binding to integrins α5β1, α_v_β_3_, or _IIb_β_3_ were −77.86, −62.57, and −65.57, respectively, which was higher than that of FN or FNIN3 (α5β1 −67.08, α_v_β_3_ −56.51, and _IIb_β_3_ −59.51).

In total, 11 AAs of FNIN2 were found to interact with 22 AAs of integrin receptor α5β1 and to form 4 H-bonds. 2 H-bonds were formed between Thr18 of FNIN2 and Thr411 of the receptor, and Gly19 and Asn8 of FNIN2 were found to interact with Gly439 and Arg122 of the receptor by H-bonding. In addition, 24 hydrophobic interactions were observed between FNIN2 and the receptor. Nine AA residues of FNIN3 interacted with 15 AAs of integrin α5β1 receptor. The FNIN 3-α5β1 interaction involved 7 hydrogen bonds and 16 hydrophobic interactions between FNIN 3 and α5β1.

**Figure 2:**
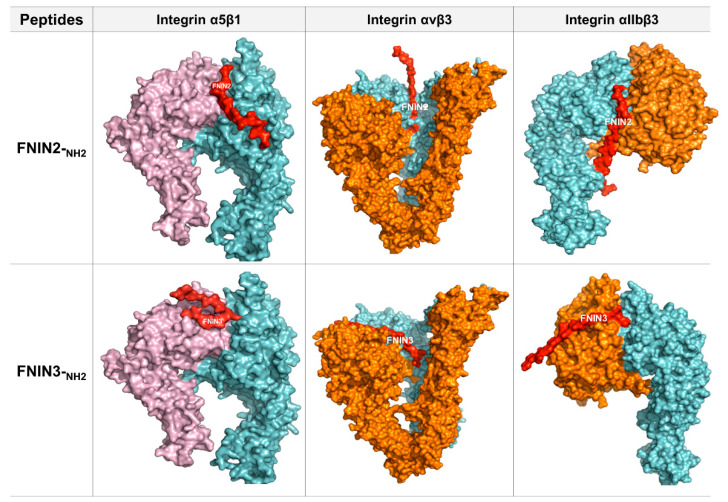
Molecular interactions between FNIN2 or FNIN3 and integrins. 3D visualization of FNIN2 or FNIN3 integrin (α5β1, αvβ3, and αIIbβ3).

Similarly, 11 AAs of FNIN2 was found to interact with 14 AAs of integrin αvβ3 through a single H-bond and 19 hydrophobic interactions. Besides, 12 AAs of FNIN3 interacted with 13 AAs of integrin αvβ3 via 5 H-bonds and 13 hydrophobic interactions. Furthermore, 17 AAs of integrin αIIbβ3 interacted with 12 AAs of FNIN2 via 5 H-bonds and 18 hydrophobic interactions; and 12 AAs of αIIbβ3 interacted with 12 AAs of FNIN3 via 3 H-bonds and 17 hydrophobic interactions (Table 1b, Figure 2, and Supplementary Figure S1).

### 2.3. Cells Proliferation Induction by FNINs

In order to determine the effect of FN on cell proliferation, the protein was treated and incubated in seventeen cells for four days. In contrast to non-treated cells, the proliferation of HeLa, HepG2, A431, Cos7, 3T3L1, Vero, C6, and MKN28 cells was substantially increased in FN protein-treated cells.

Seventeen different cell types were cultured with three different concentrations (250, 500, and 1000 nM) of FNIN2-_NH2_ and FNIN3-_NH2_ for 4 days, and cell proliferation was assessed using MTT assays. Cell proliferation was increased significantly by FNIN2-_NH2_ (for nine cell types) as compared with non-treated cells. (Table 2a). The proliferation of eight cell types was increased by FNIN3^#^. (Table 2b). The results showed that cell proliferation was influenced by FNIN concentration and cell type.

To determine the relationship between cell proliferation and the effects of FNINs, the presence of integrins α5β1, αvβ3, and αIIbβ3 in cells was searched, and results showed that the adhesion capability of HeLa cells to endothelial monolayer was significantly affected by αvβ3 integrins [27]. The proliferation of MDA-MB-231 and Hek293 cells decreased significantly after treatment with FNIN2-_NH2_ and analysis showed these cells expressed low integrin αV and αvβ3, respectively [28,29]. These reported findings indicate that the interaction between FNINs and integrin αvβ3 is critical for cell proliferation.

**Figure 3:**
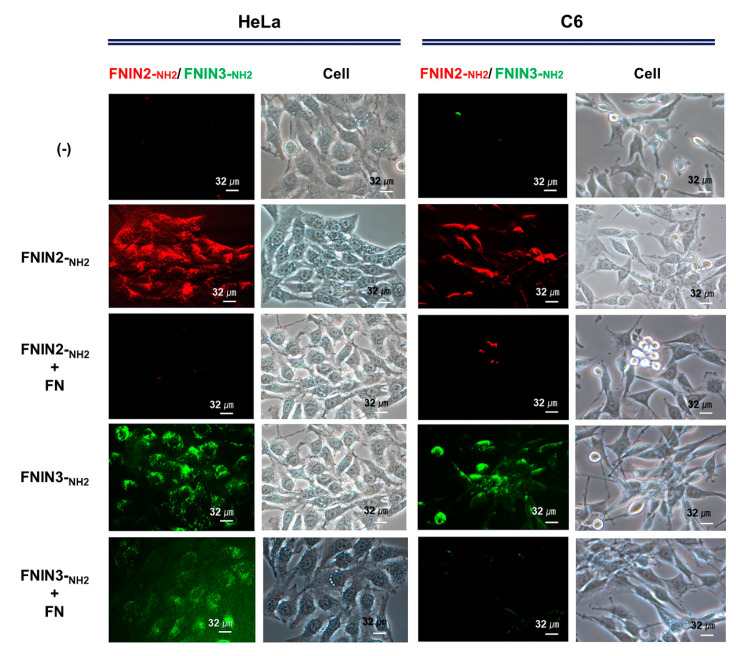
Detection of FNIN2-_NH2_ and FNIN3-_NH2_ with FN protein in HeLa and C6 cells. Rhodamine-labeled FNIN2-_NH2_ or FITC-labeled FNIN3-_NH2_ with FN protein was incubated in cultured media for 30 min, washed with PBS and observed under a fluorescence microscope.

To confirm FNIN2-_NH2_ interaction with cell lines, rhodamine-labeled FNIN2^#^ was added to cells. Rhodamine-labeled FNIN2^#^ was strongly detected on the cell membranes of HeLa, HepG2, A498, and Du145 cells while weak fluorescence was observed on the cell membranes of C2C12, MDA-MB-231, and fibroblast cells (Supplementary Figure S2). Rhodamine-labeled FNIN2-_NH2_ or FITC-labeled FNIN3-_NH2_ was added with FN protein to confirm the specific binding with HeLa and C6 cell membrane. Rhodamine-labeled FNIN2-_NH2_ and FITC-labeled FNIN3-_NH2_ were strongly detected on HeLa and C6 cells. However, rhodamine-labeled FNIN2-_NH2_ and FITC-labeled FNIN3-_NH2_ with FN protein showed weak fluorescence compared with only FNINs treated cells (Figure 3). These findings showed that the FNINs significantly interact with the cell membrane of HeLa and C6 cells.

### 2.4. Assessment of Cell Attachment and Proliferation in FNINs Coated Plate

**Figure 4:**
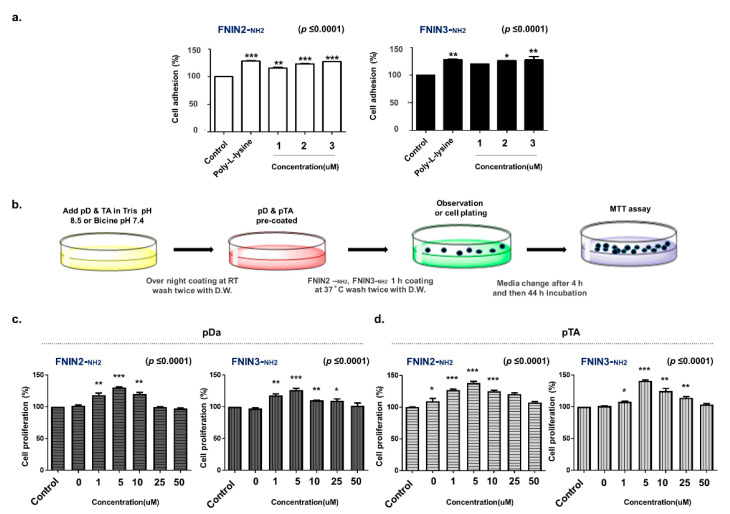
Effects of FNIN2-_NH2_ and FNIN3-_NH2_ on cell adhesion and proliferation. (**a**) HBEpiC cell adhesion on FNIN2-_NH2_, FNIN3-_NH2_, or poly-lysine coated plates. (**b**) Schematic of the coating method used. (**c**,**d**) Cell proliferations on FNIN2-_NH2_ or FNIN3-_NH2_ after pTA or pD pre-coating. Means ± SD (*n* > 3). * *p* ≤ 0.05, ** *p* ≤ 0.001, *** *p* ≤ 0.0001.

Human bronchial epithelial cells (HBEpiC cells) adhesions were increased by 15% and 3%–11%, respectively, in poly L lysine (PLL) and FNIN2-_NH2_ or FNIN3-_NH2_ (1–4 μM)-coated plates as compared with non-coated plates. The cell adhesion efficacy was lower for only FNIN-coated plates than for PLL-coated plates (Figure 4a), which was attributed to a relative lack of functional groups on FNINs. Dopamine (pD) and polymerized TA (pTA) can deposit on surfaces and easily bind to amine- or thiol-terminated compounds to form a functional coating [30,31]. Therefore, we pre-coated plates with pD or pTA to assess the cell proliferation efficacies of FNIN2-_NH2_ and FNIN3-_NH2_ (Figure 4b, Supplementary Figure S3a). To optimize the FNIN2-_NH2_ and FNIN3-_NH2_ coating, pD or pTA were precoated at different concentrations (pD: 0–20 μg/cm^2^; pTA: 0–50 μg/cm^2^), and then plates were coated with 2 μM of fluorescent-labeled FNIN2-_NH2_ or FNIN3-_NH2_ for 1 h. The maximum fluorescence intensities of pD and pTA coated plates were measured using a fluorometer and an AmiX imager (Supplementary Figure S3b,c). At these concentration levels, cell proliferation was similar to non-treated controls (Supplementary Figure S3b). However, excessive polymerization of dopamine and TA inhibited FNIN coating efficacy (Supplementary Figure S3b,c).

Double coating increased the cell proliferation of HBEpiC primary cells treated with pD^+^ FNIN2-_NH2_ (5 μg/cm^2^) or pDa^+^ FNIN3-_NH2_ (1 μg/cm^2^) by 30% and 40%, respectively, and those of pTA^+^ FNIN2-_NH2_ (20 μg/cm^2^) and pT^+^ FNIN3-_NH2_ (20 μg/cm^2^) were increased by 37% and 34%, respectively (Figure 4c,d). These results showed an increase in the proliferation of HBEpiC primary cells, indicating that FNIN2 or FNIN3 coatings on pD or pTA pre-coated plates enhance HBEpiC cell attachment.

### 2.5. Effects of FNIN2 or FNIN3 on the Proliferation and Osteogenic Differentiation of Human Mesenchymal Stem Cells (MSCs)

**Figure 5:**
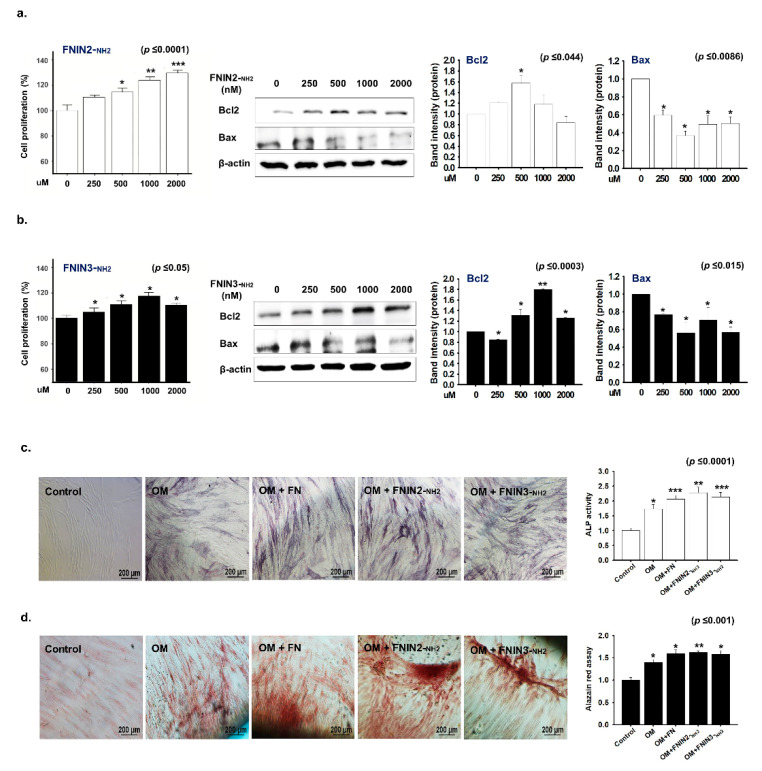
Effect of FNIN2-_NH2_ or FNIN3-_NH2_ on the proliferation and osteogenic differentiation of MSCs. Human adipose-derived mesenchymal stem cells (MSCs) were treated with different concentrations of FNIN2-_NH2_ or FNIN3-_NH2_ for 72 h and then cell proliferations and protein expressions were analyzed using a CCK-8 assay and by Western blot analysis, respectively. (**a**,**b**) MSCs proliferation and proteins (Bcl2 and bax) expression with FNIN2-_NH2_ or FNIN3-_NH2_. (**c**) Quantification of alkaline phosphatase (ALP) activity in cells cultured in osteoblast differentiation media (12 days, magnification x100, scale bar = 200 μm) (**d**) Quantification of alizarin red in cells cultured in osteoblast differentiation media (24 days, magnification x200, scale bar = 200 μm). Non-treated cells were used as controls. Means ± SD (*n* > 3). * *p* ≤ 0.05, ** *p* ≤ 0.001, *** *p* ≤ 0.0001.

The treatment of MSCs with FNIN2-_NH2_ for 72 h dose-dependently increased their proliferation (Figure 5a). Furthermore, Western blot analysis showed upregulation of Bcl2 and downregulation of Bax expressions, which further confirmed improved MSC proliferation by the peptide (Figure 5a). The treatment of MSCs with FNIN3-_NH2_ also improved their proliferation significantly (Figure 5b).

To investigate the effect of FNINs on the osteogenic differentiation of MSCs, the cells were cultured in an osteogenic medium (OM), stained, and quantified. Results showed higher alkaline phosphatase (ALP) activity in MSCs when treated with FNIN2-_NH2_ and FNIN3-_NH2_ peptides when compared to the MSCs treated with OM only (Figure 5c). Alizarin red staining revealed intense coloration, indicating mineral deposition in OM in the presence of FN or peptides (Figure 5d). In addition, a significant increase in osteogenic differentiation was observed when MSCs were treated with FNIN2-_NH2_, or FNIN3-_NH2_ as compared with MSCs treated with OM only (Figure 5d).

### 2.6. C2C12 Cell Proliferation Induced by FNIN2-_NH2_ or FNIN3-_NH2_ in Alginate Beads

**Figure 6:**
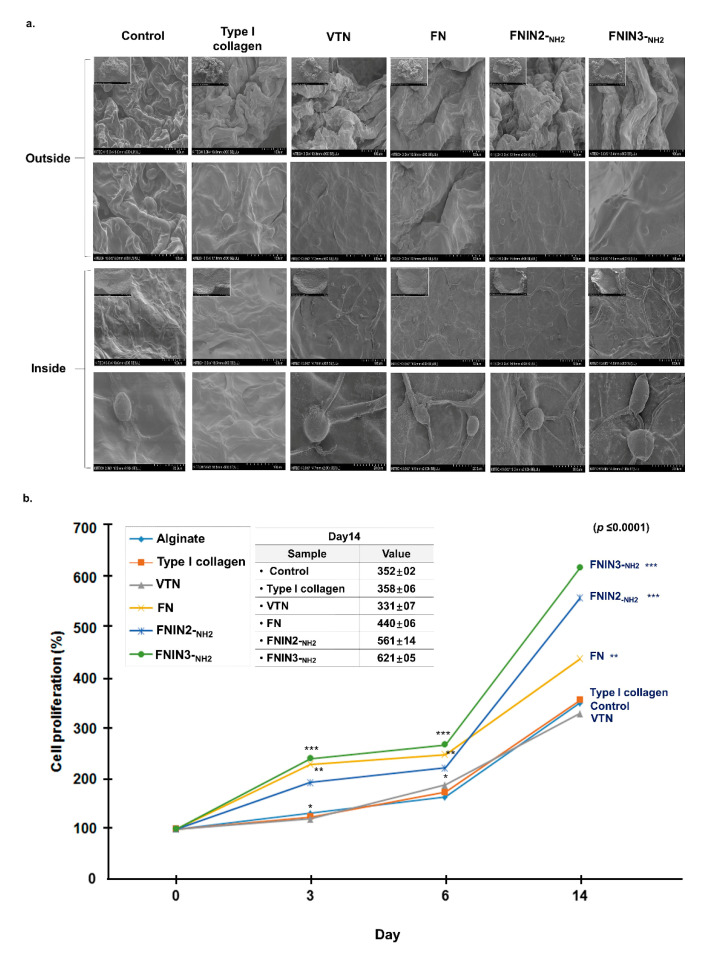
Analysis of cell proliferation in alginate beads containing FNIN2-_NH2_ or FNIN3-_NH2_ C2C12 cells were cultured for 0, 3, 6, or 14 days in collagen, VTN, FN, FNIN2-_NH2_, or FNIN3-_NH2_ containing alginate beads. Cell morphologies inside beads were determined by SEM and cell numbers were counted using a hemocytometer. (**a**) SEM images of bead. (**b**) Cell proliferations (%) for different types of ECM and FNIN treatments at different time points. Means ± SD (*n* > 3). * *p* ≤ 0.05, ** *p* ≤ 0.001, *** *p* ≤ 0.0001.

C2C12 cells in alginate beads containing collagen, vitronectin (VTN), FN, FNIN2-_NH2_, or FNIN3-_NH2_ were cultured for 0, 3, 6, or 14 days. The cell morphologies in beads were observed by SEM (Figure 6a) and cell numbers were counted. Cell proliferation was increased in the presence of type I collagen, FN, FNIN2-_NH2_, or FNIN3-_NH2_ as compared with the alginate bead cell (control) (day 14), and this was greatest for FNIN3-_NH2_. However, reduced cell proliferation was observed in cells treated with VTN as compared with cells cultured in alginate beads alone (Figure 6b). These results show that cell proliferation was significantly enhanced in FNIN-treated alginate beads.

## 3. Discussion

The ECM is a complex meshwork of proteins and occupies most of the volume between cells in tissues, and primarily provides mechanical support and communication between cells [32]. FN is a major component of ECM and is known to bind with several components of ECM and to transmembrane receptors (mostly integrins). Importantly, the biomechanical properties of ECM can influence cell behavior. Furthermore, ECM robustness is an essential property through which cells perceive external forces and respond appropriately to the environment, which is referred to as mechanotransduction [33].

A number of studies have shown ECMs impact the fate of stem cells [34,35]. The natural compositions of ECMs differ and depend on the tissue of origin. The ECM contains numerous adhesion proteins, such as collagen, FN, and laminin, which are exposed to stem cells. Such adhesion proteins attach using cell-binding epitopes to integrins on cell surfaces. These epitopes are short sequences of peptides originating from adhesion proteins, such as collagen RGD, FN RGDS, and IKVAV, and laminin sourced YIGSR [36]. A thorough understanding of this mechanism is crucial for the development of new biomaterials for stem cell studies.

Several authors have shown that the cell-binding region of FN is essential for binding with integrin and that this binding promotes cell adhesion, proliferation, and signaling pathways [37,38]. Thus, in the present study, this region (a total of 274 AAs) of FN was targeted for the in silico design of short peptides to improve cell culture. Furthermore, the well-known RGD cell-binding motif is located in this region (Figure 1). The RGD sequence of FN has been reported to bind with several integrin receptors [39,40], and some RGD-based peptides have been demonstrated to enhance cell adhesion and proliferation and to bind more strongly to most integrin types [41,42].

We selected several sets of short sequences ranging from 10–20 AAs from the cell-binding region of FN and employed certain modifications and assessed their in silico binding efficacies with α5β1, αvβ3, and αIIbβ3 and compared their binding efficacies with FN–integrin interactions. Of the several short sequences examined, four with greater efficacies than FN were synthesized and subjected to in vitro analysis, FNIN2 and FNIN3 produced the best results according to lab studies. The physicochemical properties (e.g., isoelectric point, net charge at pH 7.0, average hydrophilicity, GRAVY (grand average of hydropathy), and molecular weight) of FNIN2 and 3 were predicted using online peptide analyzing tools (Table 1a).

The global energies of FNIN2 and 3 and native FN against integrin receptors were considerably different. FNIN2 and 3 had higher global energies than FN against all three integrin receptors (α5β1, αvβ3, and αIIbβ3) (Table 1b). In-depth analysis of FNIN interactions with integrin receptors showed that some important amino acid residues of the short peptides interacted with integrin receptors (Figure 2 and Supplementary Figure S1). 2D representations of peptide–receptor complexes by Ligplot/Dimplot address peptide and peptide AA-level interactions with receptors [43]. The number of residues involved in interactions reflects the stability of interactions between ligands and receptors. FNIN2 showed the best binding efficacy (−77.86) with α5β1 receptor, as 11 AAs of FNIN2 interacted with 22 AAs of α5β via H-bond and hydrophobic interactions.

FNIN2 and FNIN3 altered the proliferation of different cell types in different ways. Table 2 shows the proliferative efficacies of all 19 cells used in this study at different FNIN and FNIN concentrations. Cell proliferation was affected by FNIN concentration and integrin type (α5β1, αvβ3, or αIIbβ3) in cells. For example, HeLa cell proliferation is less in the presence of FNIN2-_NH2_ at a concentration of 500 nM, but more at a concentration of 1000 nM.

The presence of integrin receptors was examined in all 17 cells, and integrin α5β1 and αIIbβ3 were present in all cells, but the expressions of integrin αvβ3 differed. It is well known that all αv integrins (αvβ3, αvβ1, αvβ5, αVβ6, and αVβ8) and other integrin types (viz. α5β1, and αIIbβ3) bind well with ECM ligands possessing the RGD site [19,44]. In the present study, the presence of integrin αvβ3 was found to play a critical role in cell proliferative efficiency. For example, HepG2 and Huh cells are liver carcinoma cells and express integrins α5β1 and αIIbβ3. FNIN2 increased HepG2 cell numbers but decreased Huh cell counts. Interestingly, integrin αvβ3 was found in HepG2 cells but not in Huh cells, which suggested the αvβ3 promotes cell proliferation (Table 2). Likewise, in silico interaction analysis showed FNIN2 binds to αvβ3 more strongly than FN (global energy −65.57 versus −25.27) and to αvβ3 more strongly than to α5β1 or αIIbβ3, which also suggests integrin αvβ3 promotes proliferation. The prominent fluorescence observed in rhodamine-labeled FNIN2 treated cells (HeLa, HepG2, A498, and Du145) adequately demonstrated the physical attachment of FNIN2 to cell membranes (Supplementary Figure S2).

Lack of cell attachment limits primary and stem cell studies. Although treatments with proteins or poly-L-lysine or feeding cells collagen, FN, or VTN are frequently used to grow specific cells, these are either expensive, toxic, or hard to handle. Considering the potential of FN to promote the proliferation, attachment, growth, and differentiation of most cell types and its ability to bind with different integrin receptors, we designed FNIN2 and FNIN3 to address these problems.

The present study confirms the potential of FNINs to be readily bound to primary cells. Double coating experiments (FNIN2-_NH2_ or FNIN3-_NH2_ + pDa or pTA) showed a 30–40 percent increase in HBEpiC primary cell proliferation (Figure 4), and these findings are consistent with those of previous studies. As reported by Aoshiba et al., FN provided support for the attachment and survival of human bronchial epithelial cells in a medium deficient in growth factor [45].

MSCs have been suggested to be alternative therapeutic agents for bone tissue engineering due to their potential to differentiate into bone-like cells [46]. In the bone tissue engineering field, extensive studies have been carried out to provide various biological signals to MSCs to accelerate bone formation [47,48,49]. In this regard, ECM proteins including collagen and FN have been reported to promote MSC proliferation and differentiation by enhancing interactions between cells and adhesion AA sequences [50,51]. ECM proteins have been reported to accelerate bone formation by upregulating the transcription of Runx2 via integrin receptor signaling [52].

Notably, human mesenchymal stem cells that are grown on ECMs had stiffness values that mimicked brain, muscle, or bone elastic modulus, and began to release organ-specific transcription factors and undergo tissue-specific cell fate changes to neurons, myoblasts, and osteoblasts, respectively [53]. The osteogenic differentiation of rat MSCs are highly regulated by substrate stiffness and by ECM macromolecules adsorbed on biomaterial surfaces [54].

Treatments with FNIN2 or 3 increased the proliferation of human adipose-derived MSCs and increased the expression of Bcl2 (anti-apoptotic) and decreased that of Bax (pro-apoptotic). Thus, our findings confirm a beneficial impact of FNIN peptides on MSC viability in vitro. Furthermore, increased ALP staining in osteogenic differentiation media-treated cells were augmented by the addition of native FN, FNIN2-_NH2_, or FNIN3-_NH2_, and significant enhancements in osteogenic differentiation were observed when MSCs were treated with FN, FNIN2-_NH2_, or FNIN2-_NH2_ as compared with OM treated MSCs (Figure 5).

C2C12 cells in alginate beads containing FNINs proliferated and attached to cells better than C2C12 cells in alginate beads or in alginate beads containing other ECM components viz. collagen, VTN, and FN (Figure 6). Growth in 3D cell culture more accurately represents growth in ex vivo human tissue, whereas 2D cell cultures do not well represent how cells grow or how they are affected by disease or injury. Our results show that the effects of FNINs were significant in 3D culture but not in 2D culture. Taken together, our results indicate FNIN2 and FNIN3 have a great potential for commercialization based on comparisons with natural FN whole protein.

## 4. Materials and Methods

### 4.1. In Silico Experiments

#### 4.1.1. D Structures of Fibronectin and Integrin

The crystal structure of the FN binding region was retrieved from the protein data bank (PDB) (PDB ID: 1FNF and 1FNA) together with the 3D structures of integrins known to bind with FN (α5β1 (PDB: 3VI4), αvβ3 (PDB: 4G1M and 3IJE), and αIIbβ3 (PDB: 3zdy)).

#### 4.1.2. Interactions between Fibronectin and Integrins (α5β1, αvβ3, and αIIbβ3)

Protein–protein docking simulations were conducted using the web version of PatchDock (https://bioinfo3d.cs.tau.ac.il/PatchDock/, accessed on 24 January 2021) and further refined and ranked with FireDock (http://bioinfo3d.cs.tau.ac.il/FireDock/, accessed on 24 January 2021). Integrins α5β1, αvβ3, and αIIbβ3 were used as receptors for PatchDock simulations and FN as the ligand under default complex-type settings. For each interaction, 100 predictions were produced by PatchDock and all were forwarded to FireDock to obtain the 10 best solutions based on global energy.

#### 4.1.3. Prediction of Fibronectin Short Peptides

Initially, 10–20 AA sequences were selected from the FN cell-binding region to encompass RGD- and non-RGD-based sequences. Several modifications were made in selected sequences, e.g., changes in AA positions, aa deletions, or additions, and we named these short peptides “FNINs” (fibronectin-based intergrin binding peptides). These short sequences were tested for uniqueness and physicochemical properties using various web resources and tools (https://www.nextprot.org/tools/peptide-uniqueness-checker, accessed on 24 January 2021) [55]. Based on considerations of physicochemical properties and solubility, several best candidate peptides were further screened in silico for integrin binding efficacy using PatchDock and FireDock.

#### 4.1.4. Interactions between the Designed Peptides and Integrins

The selected short sequences were converted into 3D structures and their docking interactions with integrin receptors were investigated using PatchDock and FireDock. The binding efficacies of peptides with integrin(s) (α5β1, αvβ3, and αIIbβ3) were determined and compared with that of FN-integrin. Ligplot/Dimplot, a tool that generates schematic 2D representations of protein–ligand complexes, was used to analyze AA residue interactions between peptides and integrin receptors [56]. Four peptides that bound to integrin receptors more strongly than FN as determined by global energies, were synthesized and subjected to in vitro and in vivo analysis.

### 4.2. FNIN Peptide Preparation

The four designed FNIN peptides were synthesized by Peptron (Daejeon, Korea), diluted with DW or DMSO (Sigma Aldrich, St. Louis, MO, USA), and stored at −20 °C.

### 4.3. Cell Proliferation

Seventeen cell lines were purchased from the Korean Cell Line Bank (Seoul, Korea). The cells were seeded at 1 × 10^3^ or 2 × 10^3^ cells/mL and incubated in DMEM or RPMI (Hyclone, UT, USA) both containing 10% fetal bovine serum (FBS, Hyclone) and 1% penicillin/streptomycin (P/S, Hyclone) supplemented with 250, 500 or 1000 nM of FNINs or FN protein (1000 nM) for 4 days in a 5% CO_2_ incubator at 37 °C. Cell proliferation was analyzed using an MTT assay. In brief, cells were washed with DMEM or RPMI and then incubated with 0.5 mg/mL of MTT reagent (Sigma Aldrich, St. Louis, MO, USA) for 1 hr. Formazan crystals were then dissolved in DMSO (Sigma Aldrich), and absorbances were measured at 540 nm using a spectrophotometer (Tecan Group Ltd., Männedorf, Switzerland). Cell proliferation was calculated using the following equation: cell viability (%) = sample OD/control OD × 100. Cell types and culture conditions are summarized in Supplemental Table S2.

### 4.4. Binding of FNIN2-_NH2_ Peptide to Cells

Cells were treated with rhodamine derivatized FNIN2-_NH2_ at 1000 nM in DMSO for 30 min in a humidified 5% CO_2_ incubator at 37 °C, washed with PBS, and imaged under a fluorescence microscope equipped with a digital camera (Nikon, Tokyo, Japan).

### 4.5. Binding of FNIN2-_NH2_ and FNIN3-_NH2_ Peptides with FN Protein to Cells

Rhodamine derivatized FNIN2-_NH2_ or FITC derivatized FNIN3-_NH2_ (1000 nM) was treated with FN protein (1000 nM) for 30 min in a humidified 5% CO2 incubator at 37 °C, washed with PBS, and imaged under a fluorescence microscope equipped with a digital camera (Nikon, Tokyo, Japan).

### 4.6. Polymerized Dopamine (pD) or Polymerized TA (pTA) Pre-Coating Prior to FNIN2-_NH2_ or FNIN3-_NH2_ Coating on Plates

To prepare pD (pD: 0, 1, 5, 10, 25 and 50 μg/cm^2^) and pTA (0, 10, 20, 30, 40 and 50 μg/cm^2^) pre-coated plates, dopamine and TA solution (Sigma Aldrich) in DW was added to a plate with an appropriate concentration, and then Tris (10 mM, pH 8.7) and bicine buffer (100 mM, pH 7.4) to give a final pH value of 8.5 and 7.4, respectively. Plates were incubated in each solution overnight at room temperature with stirring and were washed twice with DW. For FNINs coating, 1uM of FNIN2-_NH2_ or FNIN3-_NH2_ in DMSO or DW were added to pre-coated plates, incubated for 1 h at 37°C, and washed twice with DW. Coated plates were immediately used to observe the fluorescence or seed Human Bronchial Epithelial Cells (HBEpiC cells, BEpiCM, ScienCell, Carlsbad, CA, USA).

### 4.7. Cell Adhesion and Proliferation Analysis in HBEpiC Cells

HBEpiC cells were obtained from ScienCell (USA) and grown and maintained in bronchial epithelial cell medium (BEpiCM, ScienCell, USA) containing bronchial epithelial cell growth supplement (BEpiCGS, ScienCell, USA), 100 units/mL penicillin (Hyclone), and 0.1% 100 µg/mL streptomycin (Hyclone), in a 5% CO_2_ humidified incubator at 37 °C. Cells were seeded in 48-well FNIN2-_NH2_, FNIN3-_NH2_, or poly-L-lysine coated plates at a density of 4 × 10^4^ cells/well, grown for 4 h, and washed once with PBS to assess cell adhesion efficacy. After incubation for 44 h in fresh media, 20 μg of MTT dissolved in 20 μL of PBS was added to each well and incubated at 37 °C for 3 h. Media were carefully removed and 100 μL of DMSO was added to each well. Formazan crystals were completely dissolved with shaking in DMSO, and absorbance was measured at 540 nm. Untreated cells were used as controls. Cell proliferations were calculated using by expressing sample ODs as percentages of control ODs.

### 4.8. Proliferation of MSCs by FNIN2^#^ and FNIN3^#^

Human adipose-derived mesenchymal stem cells (MSCs) were purchased from Stemore (Incheon, Korea) and cultured in MEM-alpha modified media (Hyclone) containing 10% FBS and 1% P/S. MSCs at passage numbers 6 to 8 were used. To investigate the effects of FNIN peptides, cell proliferations were determined using a Cell Counting Kit (CCK-8, Donjindo Molecular Technologies Inc., MD, USA). Briefly, 1 × 10^4^ MSCs were cultured in a 96-well plate for 24 h and allowed to attach. Cells were then treated with FNIN2-_NH2_ or FNIN3-_NH2_ (250, 500, 1000, or 2000 nM) for 72 h, 5 μL of CCK-8 reagent was added per well, and the cells were incubated in dark for 4 h. Absorbances were read at 450 nm using SPARK 10M (TECAN, Untersbergstrasse, Grodig, Austria). In addition, results were confirmed by Western blot analysis as previously described [57]. Briefly, the peptide-treated MSCs were lysed in ice-cold RIPA buffer (Thermo Scientific, Waltham, MA, USA) containing protease and phosphatase inhibitors (Thermo Scientific) and an equal amount of proteins from each sample was separated by sodium dodecyl sulfate-polyacrylamide gel electrophoresis (SDS-PAGE). Proteins were transferred to Immobilon-P membranes (Millipore Corporation, Billerica, MA, USA), which were probed with antibodies against Bcl2 (1/1000, Cell Signaling Technology, Danvers, MA, USA) and Bax (1/1000, Cell Signaling Technology) using β-actin (1/1000, Cell Signaling Technology) as a loading control. The membranes were then treated with HRP-conjugated secondary antibody, washed three times, and blots were developed using chemiluminescence detection reagent (Thermo Scientific). The whole blot is provided in Supplementary Figure S4.

### 4.9. Osteogenic Differentiation of MSC by FNIN2-_NH2_ and FNIN3-_NH2_

MSCs were seeded in a 96-well plate at a density of 1 × 10^3^ per well, allowed to attach for 24 h, and then cultured in osteogenic medium (OM) supplemented with 0.1 mg/mL of FNIN2-_NH2_ or FNIN4-_NH2_. As a positive control, MSCs were also treated with 0.1 mg/mL of FN whole protein (Sigma Aldrich). Media were refreshed every two days with fresh media containing the respective peptides. The ALP assays and alizarin red staining were performed at day 12 or 24 to investigate the effects of peptides on the osteogenic differentiation of MSCs. The preparation of differentiation media, estimation of ALP activity, and quantification of alizarin red staining were performed as we previously described [58].

### 4.10. Alginate Bead Preparation, 3D Culture, and Cell Proliferation

Alginate powder was mixed with alginic acid sodium salt (Sigma Aldrich) and incubated at 65 °C for 1 hr. After cooling, alginate solution and C2C12 cells (1 × 10^6^ cells/mL) were mixed with type I collagen (2860 nM, Sigma Aldrich), VTN (13 nM, Sigma Aldrich), and FN (2.2 nM), FNIN2-_NH2_ (1000 nM), or FNIN3-_NH2_ (1000 nM) and then beads were made using 1% CaCl_2_ (Sigma Aldrich). Cells in alginate beads were cultured with DMEM+20%FBS+1%P/S for 0, 3, 6, or 14 days and cells were isolated by melting beads with 50 mM EDTA (Sigma Aldrich). Cell numbers were counted using a hemocytometer. To observe bead centers, alginate beads were dried using a freeze dryer (VirTis-An SP Scientific, Warminster, PA, USA), cut, and cut surfaces were coated with platinum (Pt) and observed under a scanning electron microscope (SEM, Hitachi, Tokyo, Japan).

### 4.11. Statistical Analysis

Tukey’s Studentized test was employed to analyze the mean values of cell proliferation. Image J software (National Institutes of Health, Bethesda, MA, USA, https://imagej.nih.gov/ij/, 1997–2018, accessed on 24 January 2021) was used to quantify band intensities in Western blots. Protein expressions were normalized versus β-actin (the internal control), and the analysis was conducted using one-way ANOVA and PROC GLM in SAS, ver.9.0 (SAS Institute, Cary, NC, USA).

## 5. Conclusions

Summarizing, FNIN2 and FNIN3, two new ECM mimetic peptides, were found to improve the attachments, proliferation, and differentiation of primary cells and stem cells. FNINs dramatically improved physical attachment to the membranes of HeLa, HepG2, A498, and Du145 cells. Besides, we demonstrated the cell attachment potency of FNINs using primary HBEpIC cells using FNIN coatings applied on pD or pTA precoated plates. The improved viability and osteogenic differentiation ability of human adipose-derived MSCs conferred by FNINs confirmed they improved osteogenic efficacy. In addition, FNINs were more effective in 3D than in 2D culture systems. Collectively, based on our observations and on the premise that the small sizes of FNINs as compared with natural FN infer cost-effectiveness and easier handling, we believe FNINs should be considered novel ECM mimetic biomaterials.

## Figures and Tables

**Table 1:** General properties of fibronectin-based intergrin binding peptides (FNINs) and their binding efficacies with integrins. a. Physicochemical properties of peptides. b. Binding efficacies (global energies) of integrin inactions with FN and designed peptides.

**(a)**										
**No.**	**Name**	**Sequence**	**Length**	**Gravy**	**Molecular Weight**	**M.W Monoisotopic**	**Isoelectric Point**	**Net Charge at pH7.0**	**Average Hydrophilicity**	**Ratio of Hydrophilic Residues/Total Number of Residues (%)**
1	FNIN2-_NH2_	LSISPSDNAVVLTNLLPTGE	20	0.425	2040.3	2039.0787	4.1	−1	−0.3	35
3	FNIN3_-NH2_	TVYAVTGRGDSPASSKPC	18	−0.36	1795.01	1794.8571	9.8	2	0.1	33
**(b)**										
**Integrin Type**			**FN**				**FNIN2_-NH2_**		**FNIN3_-NH2_**	
α5β1			−58.51				−77.86		−67.08	
αIIbβ3			−40.19				−62.57		−56.51	
αvβ3			−25.27				−65.57		−59.18	

**Table 2:** Impacts of FNIN2 and FNIN3 on cell proliferation. a. FNIN2-_NH2_ effects. b. FNIN3-_NH2_ effects. 0 nM treated cells were used as controls. Means ± SD (*n* > 3).

**(a)**						
**No.**	**Cells**	**FNIN2-_NH2_**				
		**0 nM**	**250 nM**	**500 nM**	**1000 nM**	***p* Value**
**1**	C2C12	100 ± 0	97 ± 3	95 ± 2	104 ± 2	0.0476
**2**	HeLa	100 ± 0	103 ± 4	90 ± 5	115 ± 7	0.0500
**3**	HepG2	100 ± 0	99 ± 1	97 ± 3	108 ± 1	0.0213
**4**	A498	100 ± 0	111 ± 5	102 ± 1	114 ± 4	0.0203
**5**	Du145	100 ± 0	100 ± 1	94 ± 1	117 ± 5	0.0003
**6**	MDA-MB-231	100 ± 0	106 ± 2	96 ± 2	91 ± 1	0.0002
**7**	MRC-5	100 ± 0	112 ± 5	101 ± 3	107 ± 1	0.0261
**8**	HT29	100 ± 0	98 ± 1	102 ± 0	111 ± 3	0.0002
**9**	A431	100 ± 0	103 ± 1	107 ± 0	102 ± 0	0.022
**10**	Fibroblast	100 ± 0	91 ± 0	88 ± 2	97 ± 11	0.5261
**11**	Cos7	100 ± 0	97 ± 4	94 ± 1	102 ± 2	0.1778
**12**	Raw246.7	100 ± 0	110 ± 2	108 ± 4	109 ± 2	0.0988
**13**	3T3L1	100 ± 0	99 ± 2	99 ± 1	102 ± 3	0.5346
**14**	Vero	100 ± 0	81 ± 2	90 ± 3	93 ± 3	0.0027
**15**	Hek293	100 ± 0	100 ± 4	81 ± 4	95 ± 6	0.0424
**16**	C6	100 ± 0	90 ± 4	94 ± 1	113 ± 2	0.0114
**17**	MKN28	100 ± 0	95 ± 1	93 ± 1	97 ± 1	0.0114
**(b)**						
**No.**	**Cells**	**FNIN3-_NH2_**				
		**0 nM**	**250 nM**	**500 nM**	**1000 nM**	***p* Value**
**1**	C2C12	100 ± 0	99 ± 1	95 ± 1	99 ± 1	0.0069
**2**	HeLa	100 ± 0	97 ± 1.4	111 ± 0.2	115 ± 3	0.0034
**3**	HepG2	100 ± 0	99 ± 6	117 ± 3	116 ± 3	0.0001
**4**	A498	100 ± 0	103 ± 0	122 ± 3	111 ± 3	0.0002
**5**	Du145	100 ± 0	95 ± 1	106 ± 1	99 ± 1	0.0005
**6**	MDA-MB-231	100 ± 0	96 ± 1	91 ± 2	96 ± 1	0.003
**7**	MRC-5	100 ± 0	90 ± 1	93 ± 0	95 ± 1	0.0001
**8**	HT29	100 ± 0	92 ± 0	95 ± 1	93 ± 1	0.0001
**9**	A431	100 ± 0	98 ± 1	104 ± 1	93 ± 1	0.0001
**10**	Fibroblast	100 ± 0	112 ± 4	92 ± 1	112 ± 2	0.0076
**11**	Cos7	100 ± 0	100 ± 0	96 ± 1	102 ± 1	0.0165
**12**	Raw246.7	100 ± 0	93 ± 3	82 ± 3	90 ± 2	0.0339
**13**	3T3L1	100 ± 0	98 ± 2	96 ± 2	98 ± 1	0.4426
**14**	Vero	100 ± 0	92 ± 2	88 ± 0	88 ± 3	0.0068
**15**	Hek293	100 ± 0	71 ± 1	94 ± 1	82 ± 1	0.0001
**16**	C6	100 ± 0	106 ± 4	109 ± 0	112 ± 7	0.329
**17**	MKN28	100 ± 0	93 ± 0	96 ± 1	89 ± 1	0.0006

## Data Availability

The data presented in this study are available in this article and the accompanying supplementary material.

## Supplementary Materials

The following are available online at https://www.mdpi.com/1422-0067/22/6/3042/s1, Supplementary Table S1. Effects of FN full protein on cell proliferation. 0 nM treated cells were used as controls. means ± SD (*n* > 3). * *p* ≤ 0.05, ** *p* ≤ 0.001, *** *p* ≤ 0.0001; Supplementary Table S2. Cell culture and proliferation condition with FNINs; Supplementary Figure S1. Amino acids interactions between FNIN2 or FNIN3 and integrins (α5β1, αvβ3, and αIIbβ3); Supplementary Figure S2. Detection of FNIN2-_NH2_ binding in cells. Cells were incubated in cultured media supplemented with rhodamine for 30 min, washed with PBS, and observed under a fluorescence microscope; Supplementary Figure S3. Improved coating efficacy of the FNIN peptide on a plate using a polymerization coating method with dopamine and tannic acid. (a) The characteristic of dopamine and tannic acid. (b) Cell proliferation and coating efficacy of FITC-FNIN-_NH2_ after pD and pTA pre-coating. Fluorescent intensity (FI) was measured with Spectral AmiX imaging analyzer (Spectral Instruments Imaging Inc., Tucson, AZ, USA) and represents FI = measured FI − FI (0). (c) The fluorescent intensity of FITC-FNIN-_NH2_ (2 μM) coating after pD and pTA coating on a black plate (non-treated plate, not for cell culture) was measured using SpectraMax Gemini EM (Molecular Devices, USA). Blank represents the same concentration of FITC-FNIN-_NH2_ without polymerization of each buffer; Supplementary Figure S4. Whole blots.
