# Supplementary material for: Identification of Novel FNIN2 and FNIN3 Fibronectin-Derived Peptides That Promote Cell Adhesion, Proliferation and Differentiation in Primary Cells and Stem Cells
Source: Int J Mol Sci. 2021 Mar 16;22(6):3042. doi: 10.3390/ijms22063042 (PMC8002551; doi:10.3390/ijms22063042)

Supplementary data

Supplementary Table S1.

No.	Cells	Full protein (%)	*p* value
1	C2C12	106±2	0.0891
2	HeLa	114±1	0.0091
3	HepG2	115±1	0.0028
4	A498	92±5	0.2567
5	Du145	104±1	0.0885
6	MDA-MB-231	103±2	0.2107
7	MRC-5	108±4	0.06
8	HT29	100±1	0.9756
9	A431	106±1	0.0257
10	Fibroblast	108±4	0.140
11	Cos7	115±0	0.001
12	Raw246.7	102±4	0.536
13	3T3L1	111±1	0.017
14	Vero	110±3	0.029
15	Hek29	106±3	0.121
16	C6	107±0	0.0000008
17	MKN28	119±1	0.0017

Supplementary Table S2.

NO	Cells	Media	Cell no.(12well)	Peptide con (nM)	Incubation day
1	C2C12	DMEM+10%FBS+1%P/S	1,000/well	250/500/1000	4
2	HeLa	DMEM+10%FBS+1%P/S	2,000/well	250/500/1000	4
3	HepG2	DMEM+10%FBS+1%P/S	2,000/well	250/500/1000	4
4	A498	DMEM+10%FBS+1%P/S	2,000/well	250/500/1000	4
5	Du145	RPMI+10%FBS+1%P/S	2,000/well	250/500/1000	4
6	MDA-MB-231	DMEM+10%FBS+1%P/S	2,000/well	250/500/1000	4
7	MRC-5	DMEM+10%FBS+1%NEAA+1%L-glutamine+1%Antianti	2,000/well	250/500/1000	4
8	HT29	DMEM+10%FBS+1%P/S	2,000/well	250/500/1000	4
9	A431	DMEM+10%FBS+1%P/S	2,000/well	250/500/1000	4
10	Fibroblast	DMEM+10%FBS+1%P/S	2,000/well	250/500/1000	4
11	Cos7	DMEM+10%FBS+1%P/S	2,000/well	250/500/1000	4
12	Raw246.7	DMEM+10%FBS+1%P/S	2,000/well	250/500/1000	4
13	3T3L1	DMEM+10%FBS+1%P/S	2,000/well	250/500/1000	4
14	Vero	DMEM+10%FBS+1%Antianti	2,000/well	250/500/1000	4
15	Hek29	DMEM+10%FBS+1%P/S	2,000/well	250/500/1000	4
16	C6	DMEM+10%FBS+1%P/S	2,000/well	250/500/1000	4
17	MKN28	DMEM+10%FBS+1%P/S	2,000/well	250/500/1000	4

Supplementary Figure S1.

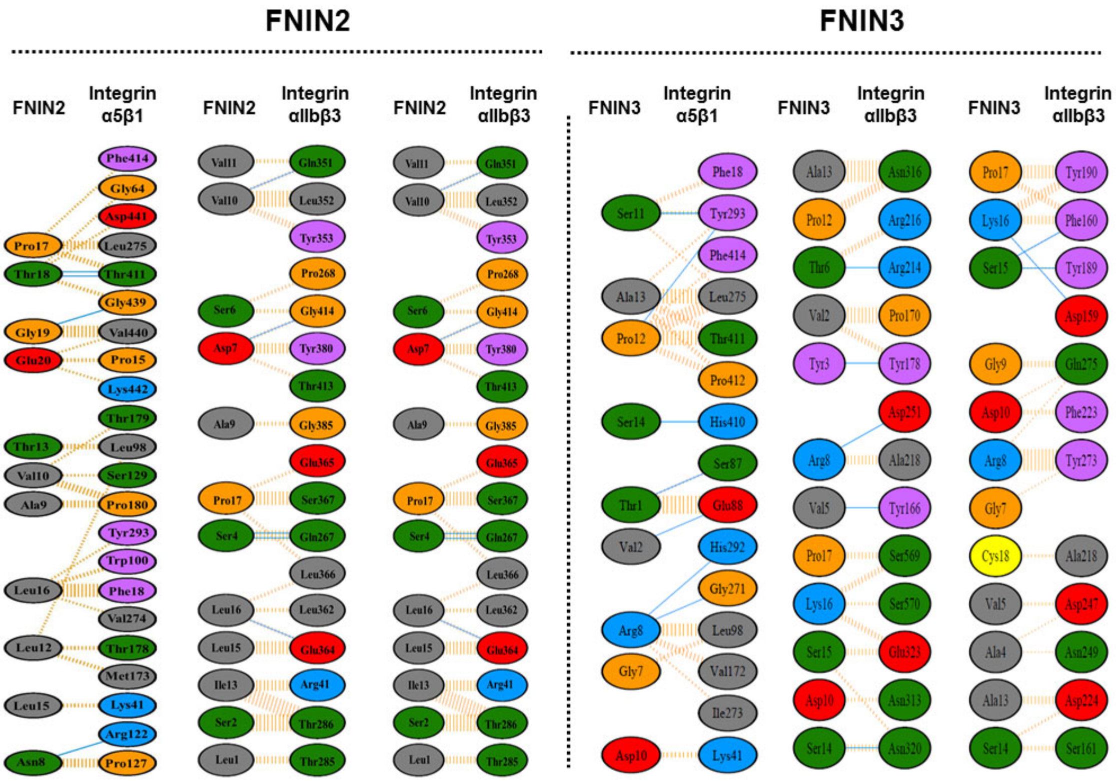

Supplementary Figure S2

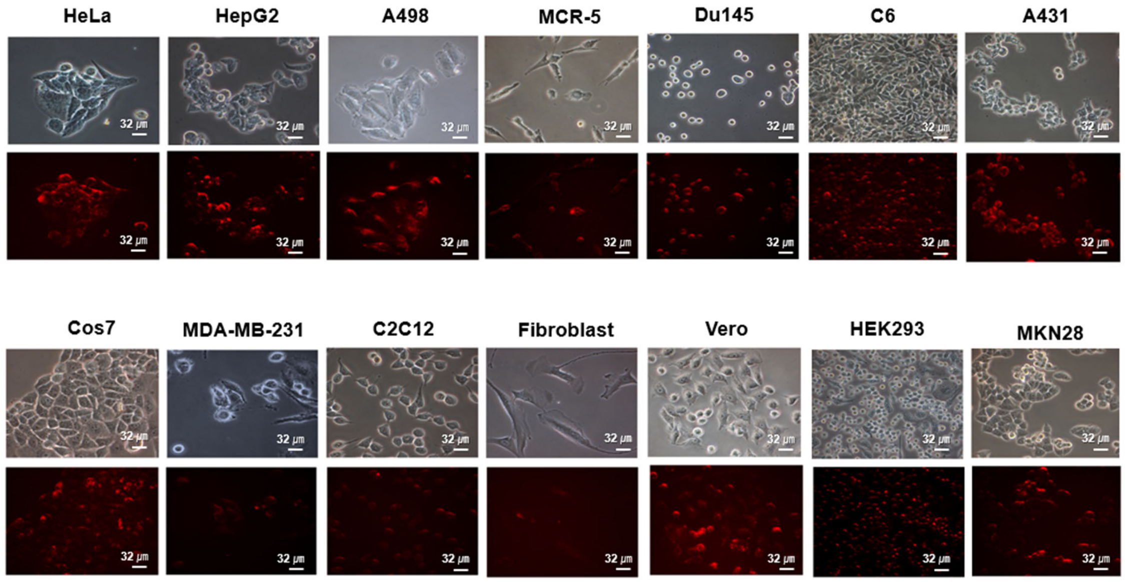

Supplementary Figure S3.

a

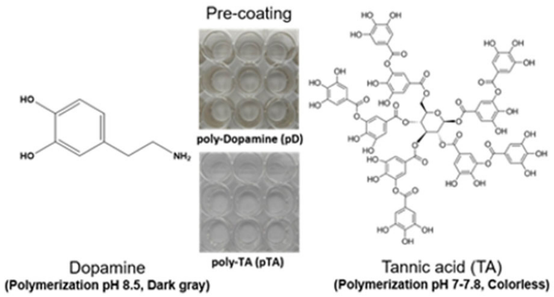

b

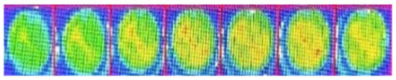

pD con. (μg/㎠)	0	0.25	0.5	1	5	10	20
FI ($x10^8$)	0	2.1	3.7	4.8	4.1	5.3	3.4
Cell Proliferation (%)	77 ±1.3	78 ±5.2	90 ±6.7	104 ±10.0	82 ±8.7	91 ±0.6	89 ±3.3

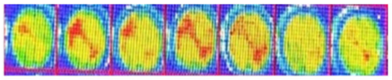

pTA con. (μg/㎠)	0	5	10	20	30	40	50
FI ($x10^8$)	0	2.7	2.5	4.0	4.4	2.5	0.6
Cell Proliferation (%)	84 ±8.3	109 ±1.3	95 ±5.0	108 ±9.8	111 ±8.5	109 ±7.9	100 ±5.4

c

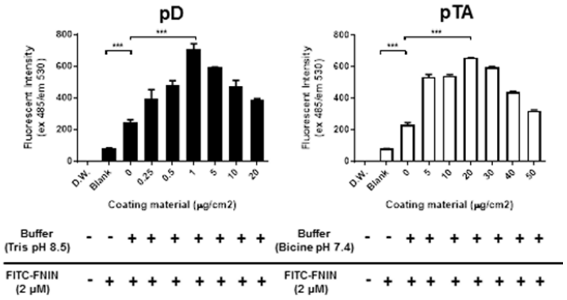

Supplementary Figure 4.

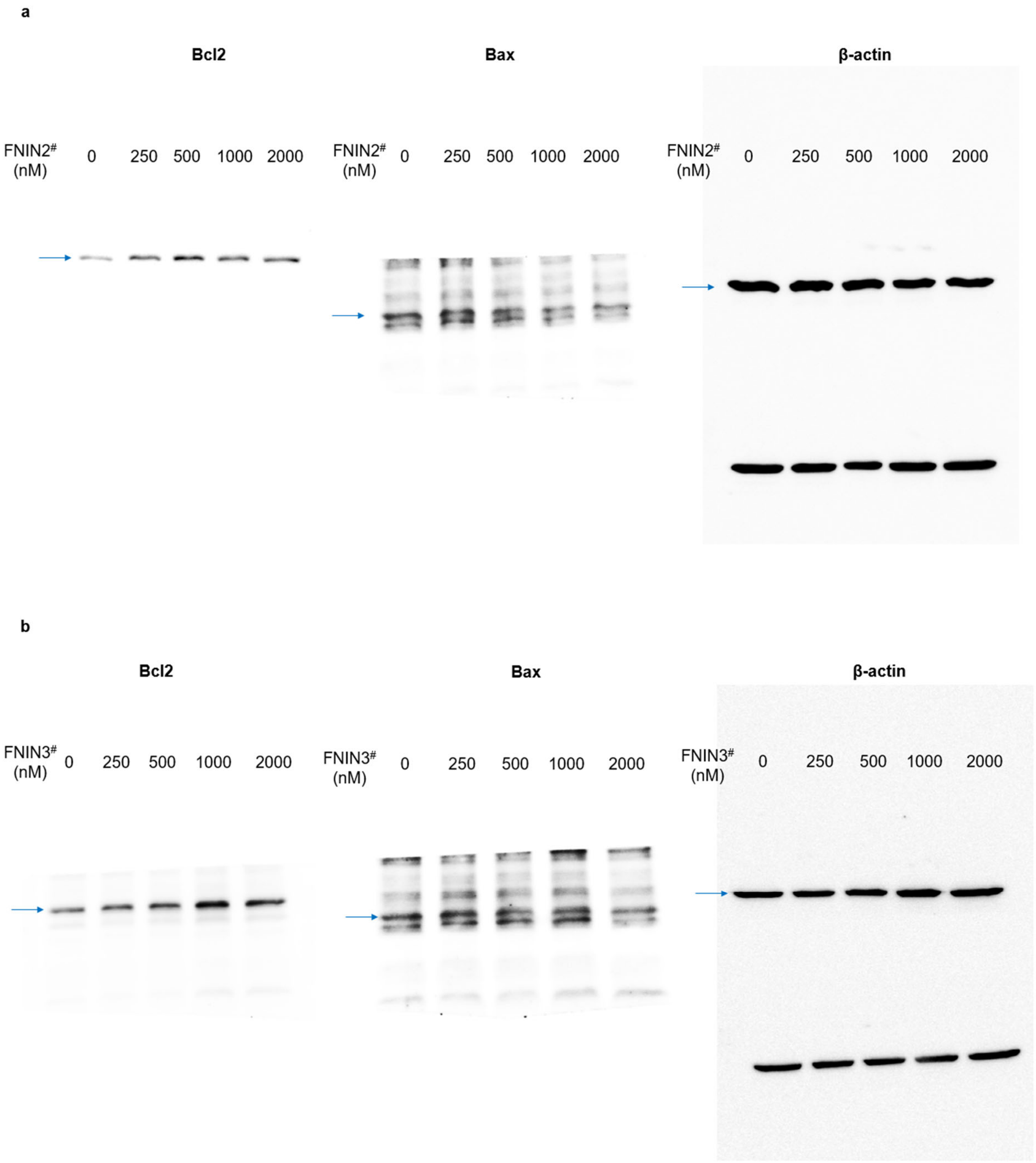

Supplement: Supplementary file 1 [file ijms-22-03042-s001.pdf]
